# Multimodal MRI reveals consistent basal ganglia and limbic system alterations in COVID-19 survivors

**DOI:** 10.1162/IMAG.a.1027

**Published:** 2025-11-26

**Authors:** Sapna S. Mishra, Caterina A. Pedersini, Rohit Misra, Preeti Yadav, Rakibul Hafiz, Bas Rokers, Bharat Biswal, Tapan K. Gandhi

**Affiliations:** Department of Electrical Engineering, Indian Institute of Technology Delhi, New Delhi, India; Psychology, Division of Science, New York University Abu Dhabi, Abu Dhabi, United Arab Emirates; Center for Brain and Health, New York University Abu Dhabi, Abu Dhabi, United Arab Emirates; Department of Biomedical Engineering, New Jersey Institute of Technology (NJIT), Newark, NJ, United States

**Keywords:** post-COVID syndrome (PCS), T1-weighted MRI, diffusion-weighted MRI (dMRI), resting-state functional MRI (rs-fMRI), arterial spin labeling (ASL)

## Abstract

The long-term impact of COVID-19 on the brain is multifaceted, encompassing structural and functional disruptions. A cohesive theory of the underlying mechanisms of the Post-COVID Syndrome (PCS) remains unknown, primarily due to high variability in findings across independent studies. Here, we present a multimodal, cross-sectional MRI analysis of brain morphology (T1-MRI), tissue microstructure (diffusion-MRI), functional connectivity (functional-MRI), and cerebral blood flow (arterial spin labeling MRI) in COVID-recovered patients (CRPs, N=76) and healthy controls (HCs, N = 51). Although the global brain volumes did not differ between the two groups, CRPs showed focal atrophy in the right basal ganglia and limbic structures, along with cortical thinning in paralimbic regions (prefrontal cortex, insula) (*p* < 0.05). Diffusion MRI analysis revealed reduced fractional anisotropy and elevated radial diffusivity in the uncinate fasciculus and cingulum. No differences were observed in resting-state functional connectivity (RSFC) and cerebral blood flow between HCs and CRPs (*p* > 0.05). We further investigated the effect of infection severity by stratifying the CRPs into hospitalized (HP; N = 21) and non-hospitalized (NHP; N = 46) groups. The microstructural damage was linked to infection severity, more pronounced in the HPs (*p* < 0.05). In HPs, RSFC was diminished between components of the default mode network and the insula and caudate as compared with HCs and NHPs (*p* < 0.05). Results suggest COVID-19 is associated with selective structural and functional alterations in basal ganglia–limbic–cortical circuits, with stronger effects in severe cases. Overall, our findings both validate previously reported neuroimaging biomarkers and reveal new ones associated with the post-COVID syndrome, motivating future hypothesis-driven studies on behavioral correlates and therapeutic interventions.

## 1 Introduction

Coronavirus Disease 2019 (COVID-19) primarily manifests as a respiratory infection, characterized by common symptoms such as fever, cough, and fatigue (Cooke & Shapiro, 2003). Since its emergence in December 2019, COVID-19 has precipitated a global pandemic with profound health, economic, and societal consequences. Healthcare systems worldwide were overwhelmed, economic activities disrupted, and daily life profoundly altered through public health interventions such as mask mandates, social distancing, and widespread vaccination campaigns. Although the respiratory symptoms of COVID-19 typically resolve within weeks, mounting evidence indicates that a substantial proportion of survivors experience persistent cognitive and behavioral symptoms even after virological recovery (Badenoch et al., 2022). Reports of enduring “brain fog”, memory deficits, inattention, cognitive fatigue, and emotional disturbances have continued to surface months to years after the initial infection (Badenoch et al., 2022; Nalbandian et al., 2023). These long-term manifestations, often grouped under the umbrella of Post-COVID Syndrome (PCS) or Long COVID, have raised major public health concerns due to their impact on survivors’ quality of life and functional independence.

A growing body of literature underscores the diversity and prevalence of these sequelae. Meta-analyses indicate that approximately 32% of COVID-19 survivors report significant fatigue 12 weeks after recovery (Ceban et al., 2022), while about 22% exhibit measurable cognitive impairment. Sleep disturbances (27.4%), fatigue (24.4%), cognitive deficits (20.2%), anxiety (19.1%), and post-traumatic stress (15.7%) have emerged as the most common persistent symptoms in post-COVID cohorts (Badenoch et al., 2022). Furthermore, cognitive performance studies have shown that COVID-19 survivors display significantly lower cognitive function than uninfected controls (Cheetham et al., 2023).

Neuroimaging has played a critical role in delineating the structural, microstructural, and functional brain alterations associated with COVID-19 recovery. Earlier studies on structural MRI revealed variable tissue changes. Niu et al. (2025) reported increased gray-matter volumes in the posterior cingulate cortex and isthmus regions, whereas Douaud et al., analyzing UK Biobank data, found cortical thinning in the orbitofrontal cortex and parahippocampal gyrus following COVID-19 infection (Douaud et al., 2022). Atrophy in the orbitofrontal cortex and the olfactory bulb was also reported by Perlaki et al. (2024). More recent MRI studies have also reported reduction in gray-matter volume (GMV) in COVID-19 survivors. Cataldo et al. (2024) reported reduced GMV in the lingual gyrus, inferior parietal cortex, postcentral gyrus, precuneus, and the cerebellum. Reduced GMV in a similar set of regions was also reported by Capelli et al. (2024). Multiple studies have consistently reported gray-matter atrophy in subcortical regions such as the thalamus, putamen, pallidum, amygdala, and caudate, hinting at damage in the limbic system and basal ganglia (Capelli et al., 2024; Heine et al., 2023; Jin et al., 2024). Alongside gray-matter changes, diffusion-weighted MRI studies have uncovered widespread white-matter abnormalities. Increased mean diffusivity (MD) and reduced fractional anisotropy (FA) were observed globally in COVID-19 survivors (Arrigoni et al., 2024). Hosp et al. (2024) also reported widespread changes in white-matter microstructure of COVID-19 survivors, measured by extra-neurite volume fraction and free-fluid fraction. Meanwhile, other studies have observed tract-specific reductions in MD within the uncinate fasciculus (UF), corpus callosum, forceps minor, fronto-occipital fasciculus (FOF), superior longitudinal fasciculus, splenium, and forceps major (Dıez-Cirarda et al., 2023; Pacheco-Jaime et al., 2025).

Functional MRI studies have further demonstrated that COVID-19 is associated with altered intrinsic brain connectivity. Leitner et al. (2024) identified impaired functional connectivity (FC) between the thalamus and cortical regions, including the precentral gyrus, supramarginal gyrus, and anterior cingulate cortex. Reductions in FC within canonical networks such as the default mode network (DMN), dorsal attention network, and somatomotor network have also been reported in COVID-19 recovered patients (Paolini et al., 2023; Voruz et al., 2023), suggesting that functional network changes may be linked to persistent cognitive and emotional deficits. In many cohorts, alterations in the functional connectivity have been observed to show significant correlation with cognitive impairment, memory issues, and fatigue in COVID-19 survivors (Fineschi et al., 2024; Heine et al., 2023; Jin et al., 2024; Madden et al., 2025). Complementary studies using arterial spin labeling MRI have suggested cerebral blood flow (CBF) alterations in COVID-19 survivors. Kim et al. (2023) identified CBF reductions in the thalamus, orbitofrontal cortex, and basal ganglia, whereas Qin et al. (2021) reported decreased CBF in the superior medial frontal gyrus and insula. In contrast, another study reported hypoperfusion in the frontal, parietal, and temporal cortices in COVID-19 survivors (Ajčević et al., 2023). These vascular findings suggest that microvascular dysfunction may be associated with the pathogenesis of post-COVID neurological symptoms.

Taken together, existing studies highlight that COVID-19’s neurological impact is multifaceted, reflected in vascular, structural, and functional disruptions. However, a large section of post-COVID studies focuses on these aspects in isolation. Diversity in their findings obstructs attempts to form a cohesive theory of the mechanisms behind PCS and to identify the key brain regions or systems that are targeted by PCS. Multimodal neuroimaging approaches are, therefore, essential for the comprehensive characterization of these sequelae, and large-scale, well-powered investigations are needed to form an integrated understanding of PCS pathophysiology. Addressing this need, here we present a multimodal, cross-sectional MRI analysis of brain morphology, tissue microstructure, functional connectivity, and cerebral blood flow in COVID-19 survivors compared with healthy controls. Conducted in India, this large-scale study utilized T1-weighted MRI, diffusion-weighted MRI (dMRI), resting-state functional MRI (rs-fMRI), and ASL perfusion imaging. We first contrast COVID-19 recovered patients (CRPs) and healthy controls (HCs) across all modalities. We then stratify CRPs based on hospitalization status to explore the effects of disease severity. Finally, we synthesize findings across imaging techniques to propose mechanistic pathways linking brain injury with post-COVID symptomatology. Through this comprehensive investigation, we aim to contribute novel insights into the neural substrates of Long COVID.

## 2 Methods

### 2.1 Subject recruitment

This cross-sectional study is based on two cohorts of subjects: (i) COVID-recovered patients (CRPs) and (ii) healthy controls (HCs). The recruitment of CRPs was done using a database of 2358 COVID-19 patients who were treated at a local hospital. Among these patients, 22% needed continuous positive airway pressure (CPAP), 40% received oxygen therapy, and 14% required intubation. After 2 weeks of testing PCR negative, 100 hospitalized patients from this database were contacted for this study. In addition, 126 CRPs who did not require hospitalization were also contacted.

The inclusion criteria for the CRP cohort were (i) age above 18 years, (ii) diagnosis of COVID-19 (RT-PCR positive), and (iii) subsequent proof of recovery from infection (RT-PCR negative) within 6 months before the scan. Similarly, inclusion criteria for the HC cohort were (i) age above 18 years and (ii) no history of COVID-19 infection as on scan date (symptomatic subjects required a negative RT-PCR report to confirm). For both cohorts, exclusion criteria were a history of (i) neuropsychiatric or neurological disorders, (ii) brain surgery/trauma, or (iii) underwent ventilation during the infection (for CRPs). A COVID-19 patient with a prior laboratory-confirmed SARS-CoV-2 infection (positive RT-PCR test) was considered to have “recovered” when they had completed the prescribed isolation period with a negative RT-PCR. Additionally, CRPs were required to be free of fever, acute respiratory distress, or need for supplemental oxygen at the time of enrollment. In total, we collected MRI scans of 76 CRPs (32.38 ± 11.52 years, 19 Female) and 51 HCs (31.98 ± 8.97 years, 12 Female). All CRPs were scanned within 3 to 6 months (12–24 weeks) of recovery from the COVID-19 infection. The data were acquired during January 2021–September 2021, when vaccination in India had only recently begun and coverage was limited. So, for this cohort, it is reasonable to assume that the recruited patients were likely unvaccinated at the time of infection. Later, certain MRIs had to be excluded from specific analyses due to poor image quality (details discussed in the respective section). Additionally, 67 CRPs (31.12 ± 10.78 years, 16 Female) consented to sharing their treatment history, and 59 CRPs (30.85 ± 10.52 years, 15 female) also reported the post-COVID symptoms experienced by them after a negative RT-PCR test.

Data collection occurred under the purview of the Indian Institute of Technology Delhi, and all imaging procedures were conducted at Mahajan Imaging Center, New Delhi, in accordance with the institute review board (IRB) regulations. The pilot study was approved by the ethics committee of the Mahajan Imaging Center, and the entire study was approved by the institute ethics committee, IIT Delhi. All subjects provided informed consent before any behavioral or physical data were collected.

### 2.2 Classification based on infection severity

Among the 76 CRPs included in this study, 67 consented to share their treatment history. We used the information from their treatment history to categorize them as Non-Hospitalized Patients or Hospitalized Patients, depending on whether they needed to be hospitalized during the treatment of their COVID-19 infection. In this study, we interpret the hospitalization of a COVID-19 patient as a mark of severe infection. Subjects categorized as HPs needed procedures such as CPAP, oxygen therapy, or continuous monitoring during their treatment. However, the COVID-19 symptoms of NHPs included fever, cough, loss of taste, loss of smell, and weakness, and they did not require hospitalization for treatment. Based on these criteria, 21 (39.48 ± 11.95 years, 6F) subjects were categorized as Hospitalized Patients (HPs) and the remaining 46 (27.30 ± 7.72 years, 10F) as Non-Hospitalized Patients (NHPs). The NHP and HP groups were used to test the relationship of MRI-derived measures with infection severity.

### 2.3 Magnetic resonance imaging

T1-weighted images were collected using a 3T GE Discovery MR 750w scanner, employing the fast BRAVO sequence in 3D imaging mode with a 32-channel head coil. Imaging parameters were 12 flip angle, 450 ms inversion time (TI), 256 mm×256 mm field of view (FOV), 1.00 mm slice thickness, 152 sagittal slices, and voxels with 1 mm isotropic resolution.

dMRI scans were acquired with a spin-echo echo-planar imaging (EPI) sequence with the following parameters: 16000 ms repetition time (TR), 79.6 ms echo time (TE), 256×256×78 matrix, 2 mm slice thickness, $1\times 1\times 2\;mm^{3}$ voxel resolution. The sequence used 30 diffusion encoding directions with anterior-posterior phase encoding and $b=1000\;s/mm^{2}$ for each direction. Four T2 volumes were also acquired with no diffusion encoding $(b=0\;s/mm^{2}).$

The rs-fMRI scans were acquired using gradient echo planar imaging (EPI) with TR​/​ ​​TE=2000ms​/​30ms , 90 flip angle, 38 slices with 3 mm thickness, field of view (FOV) of $240\times 240\;mm^{2}$, matrix size of 64×64 , and voxel size of $3.75\times 3.75\times 3\;mm^{3}$. For each subject, the duration of the rs-fMRI scan was 800 s (13 min, 20 s). The subjects were instructed to remain as still as possible during the resting-state scans with their eyes open.

The perfusion MRI scans were acquired using the 2D EPI pseudo continuous arterial spin labeling (PCASL) sequence with online contrast generation between the control and tagged volumes. The FOV was set to $240\times 240\;mm^{2}$ with 42 slices of 4 mm thickness. The voxel size was $1.88\times 1.88\times 4\;mm^{3}$ and TR​/​TE=4.728 s​/​10.70 ms . An inversion time of 2.975 s was used with a labeling duration of 1.45 s and a post-labeling delay of 1.525 s.

### 2.4 Analysis of brain morphology

T1-weighted MRIs were utilized to study the morphological characteristics of brain anatomy, such as volume and tissue thickness. After a quality check of the images, we included the T1-weighted MRI of 75 CRPs (32.12 ± 11.37 years, 18 female) and 50 HCs (31.68 ± 8.79 years, 12 female) in this analysis.

#### 2.4.1 Preprocessing

The T1-weighted anatomical MRIs were processed using Freesurfer (Fischl, 2012) to remove the skull and non-brain tissue (skull-stripping). Further, to derive morphological features from the structural MRIs, we used a combination of Freesurfer and FSL (Smith, 2002) algorithms.

#### 2.4.2 Whole-brain morphological features

We used FMRIB’s Automated Segmentation Tool (FAST) to extract whole-brain morphological features such as total intracranial volume (TIV), gray-matter volume (GMV), white-matter volume (WMV), and cerebrospinal fluid volume (CSFV) (Zhang et al., 2001). We also defined the total brain tissue volume (BTV) as the TIV without including the volume of ventricles. In addition, the GMV, WMV, and CSFV were also normalized by the TIV to eliminate the confound of head size. All the features were quantile normalized to aid comparison between groups.

#### 2.4.3 ROI-based morphological features

Cortical surface reconstruction was performed using Freesurfer 6.0.0 to get the morphological features at the level of regions of interest (ROIs) in the cortex. These features include cortical volume and cortical thickness of 68 ROIs defined using the Desikan–Killiany atlas (Desikan et al., 2006) along with the mean of each hemisphere. Volume of subcortical ROIs in the brain was also extracted using the FMRIB’s Integrated Registration and Segmentation Tool (FIRST) algorithm in the FSL Toolbox. The volumes of all cortical and subcortical ROIs were normalized by the whole-brain volume (TIV) for comparison across subjects. All the cortical and sub-cortical features were quantile normalized to aid comparison between groups.

#### 2.4.4 Surface-based morphometry (SBM)

Cortical reconstruction using FreeSurfer 6.0.0, the “recon-all” processing pipeline was utilized to extract surface-based morphological features from T1-weighted MRI scans (Dale et al., 1999; Fischl et al., 1999). The individually reconstructed surfaces were inflated and mapped to a common spherical coordinate system, “fsaverage”, facilitating precise inter-subject alignment. A map of cortical thickness (CT) was obtained by calculating the distance between the pial and white-matter surfaces at each vertex (Fischl & Dale, 2000). Additionally, a cortical volume (CV) map was derived by integrating cortical thickness and surface area measurements, quantifying regional variations in GM architecture (Winkler et al., 2012). These CT and CV maps were derived for all subjects and compared with the study of the granular changes in brain morphology.

#### 2.4.5 Statistical analysis

We used non-parametric tests for statistical comparison of feature distributions due to potential violations of normality assumptions. Specifically, the two-sided Mann–Whitney U test was used as a non-parametric alternative to the two-sample t-test for group comparisons. Features within each domain were tested together and corrected for multiple comparisons using the Benjamini–Hochberg False Discovery Rate (FDR-BH) method, with statistical significance set at $p_{\text{FDR}}<0.05$ . Whole-brain morphological features, including TIV, BTV, GMV, WMV, CSFV, and their TIV-normalized variants, were assessed collectively. Volumes of subcortical ROIs (N=15 ), cortical ROIs (N=70 ), and cortical thickness values (N=70 ) were similarly compared using Mann–Whitney U tests and corrected using FDR-BH.

For SBM, a generalized linear model (GLM) was applied to compare CT and CV maps between HCs and CRPs, incorporating age and sex as covariates of no interest. Cluster-level statistical significance was determined using FDR correction at $p_{\text{FDR}}<0.05$ ($p_{unc}<0.001$ ) to account for multiple comparisons across surface vertices.

#### 2.4.6 Relationship with infection severity

T1-weighted MRIs of 50 HCs (31.68 ± 8.79 years, 12 female), 20 HPs (38.85 ± 11.90 years, 5 female), and 46 NHPs (27.30 ± 7.72 years, 10 female) passed the quality checks and were included in this analysis. To investigate the effect of infection severity on whole-brain and ROI-based morphological measures, we performed non-parametric Kruskal–Wallis H-test for each set of measures. The features were grouped identically as described for the cohort comparison. The *p*-values were corrected for FDR using the BH method at $p_{FDR}<0.05$ . Further, for measures that showed a significant effect of severity, post hoc tests were performed. Dunn’s test was utilized for the post hoc pairwise comparison of groups with multiple comparison corrections for FDR using the BH method at $p_{FDR}<0.05$ .

Similar to the comparison of HCs and CRPs, a GLM was used to study the effect of severity on the CT and CV maps with a covariate each for HCs, NHPs, and HPs. Again, age and sex were added as covariates of no interest. Significant clusters were identified using an FDR-corrected threshold of $p_{FDR}<0.05$ ($p_{unc}<0.001$ ).

### 2.5 Analysis of white-matter microstructure

Microstructural properties of the white-matter tissue were studied using dMRI. However, after a quality check, we excluded 4 CRPs and 1 HC due to poor image quality and finally included 72 CRPs (32.17 ± 11.51 years, 17F) and 50 HCs (31.68 ± 8.79 years, 12F) in this analysis. The diffusion tensor model was used to derive measures of microstructural integrity such as Fractional Anisotropy (FA), Mean Diffusivity (MD), Axial Diffusivity (AD), and Radial Diffusivity (RD).

#### 2.5.1 Preprocessing

The MRtrix3 software was employed to denoise the dMRI scans, followed by the removal of phase-encoding-induced warping, and Gibbs’ ringing artifacts (Tournier et al., 2019). The susceptibility-induced distortion artifacts were removed using a combination of the Synb0-DISCO algorithm (to synthesize images with opposite phase encoding) (Schilling et al., 2019) and FSL’s TOPUP algorithm (Andersson et al., 2003). Further, head motion and Eddy current correction were performed using FSL. VistaSoft software (https://github.com/vistalab/vistasoft) for MATLAB was employed for diffusion tensor reconstruction using the “dtiInit” function. dMRI scans were registered to the ACPC-aligned native T1 space along with a corresponding rotation of gradient vectors. The diffusion tensors were estimated using a least-squares algorithm, bootstrapped 500 times.

#### 2.5.2 Whole-brain tractography

For tracing the white-matter pathways in dMRI, we performed whole-brain deterministic tractography using the Automated Fiber Quantification (AFQ) algorithm in MATLAB (Yeatman et al., 2012). The algorithm was seeded in the white matter and used a fourth-order Runge–Kutta integration method with a 1 mm step size for tracking. The stopping criteria were set to (i) FA < 0.2 or (ii) turning angle > 30. After tractography, the algorithm also performed tract segmentation and fiber tract identification using a probabilistic atlas. Overall, 20 white-matter tracts were identified in each subject. To quantify white-matter microstructural integrity, diffusion metrics FA, AD, MD, and RD were determined for each tract.

The 20 tracts included the left and right Anterior Thalamic Radiation (ATR), Corticospinal Tract (CST), Cingulum Cingulate (CC), Cingulum Hippocampus (CH), Inferior Fronto-Occipital Fasciculus (IFOF), Inferior Longitudinal Fasciculus (ILF), Superior Longitudinal Fasciculus (SLF), Uncinate Fasciculus (UF), Arcuate fasciculus (AF), along with major and minor Callosum Forceps (CF).

#### 2.5.3 Statistical analysis

The diffusion measures (FA, MD, AD, RD) of 20 white-matter tracts were compared across the two cohorts without the assumption of normality using the Mann–Whitney U test (N = 80). FDR was controlled using the FDR-BH method at $p_{FDR}<0.05$ .

#### 2.5.4 Relationship with infection severity

dMRI scans of 50 HCs (31.68 ± 8.29 years, 12F), 20 HPs (38.85 ± 11.90 years, 5F), and 44 NHPs (27.36 ± 7.86 years, 9F) passed the quality check and were included in this analysis. To study the effect of infection severity on the diffusion measures (FA, MD, AD, RD) of 20 tracts, we conducted Kruskal–Wallis H-tests. Multiple-comparison correction was performed using the FDR-BH method at $p_{FDR}<0.05$ . For tracts that showed a significant effect of severity, post hoc comparisons between groups relied on Dunn’s Test with multiple comparison corrections for FDR using the BH method at $p_{FDR}<0.05$ .

### 2.6 Analysis of brain function

We studied functional connectivity using the resting-state fMRI scans. From the total set of subjects, we excluded 16 CRPs and 4 HCs due to poor-quality images and motion artifacts, leading to a sample size of 61 CRPs (32.02 ± 11.01 years, 14F) and 47 HCs (32.30 ± 9.23 years, 11F) for this study. We utilized Independent Components Analysis (ICA) to extract maps of functionally connected regions in the brain.

#### 2.6.1 Preprocessing

The fMRI data were preprocessed using the Statistical Parametric Mapping (SPM) toolbox in MATLAB (The MathWorks, Inc., Natick, MA, USA). The volumes were corrected for head motion using six-parameter rigid body transformations to align them to the mean functional image. Subjects with a frame-wise displacement of greater than 2 mm in any slice of the run were excluded from the study. The mean image of the motion-corrected functional volumes was co-registered to the anatomical (T1-w) image. The anatomical images were segmented to extract gray-matter (GM), white-matter (WM), and cerebrospinal-fluid (CSF) tissue probability maps (TPMs) along with a population average anatomical template using DARTEL (Ashburner, 2007). The TPMs were non-linearly warped to the MNI standard template. Subsequently, the co-registered functional volumes were normalized to the MNI space. Spatial smoothing was done using a Gaussian kernel with FWHM=8 mm . The WM and CSF nuisance signals were extracted. The nuisance signals, along with the six motion parameters, were regressed out from the fMRI time series. Temporal filtering was performed using a Butterworth band-pass filter with cut-offs at 0.01 Hz and 0.1 Hz. The functional images were then resampled to an isotropic resolution of 3 mm.

#### 2.6.2 Independent component analysis of rs-fMRI

**Fig. 1.**
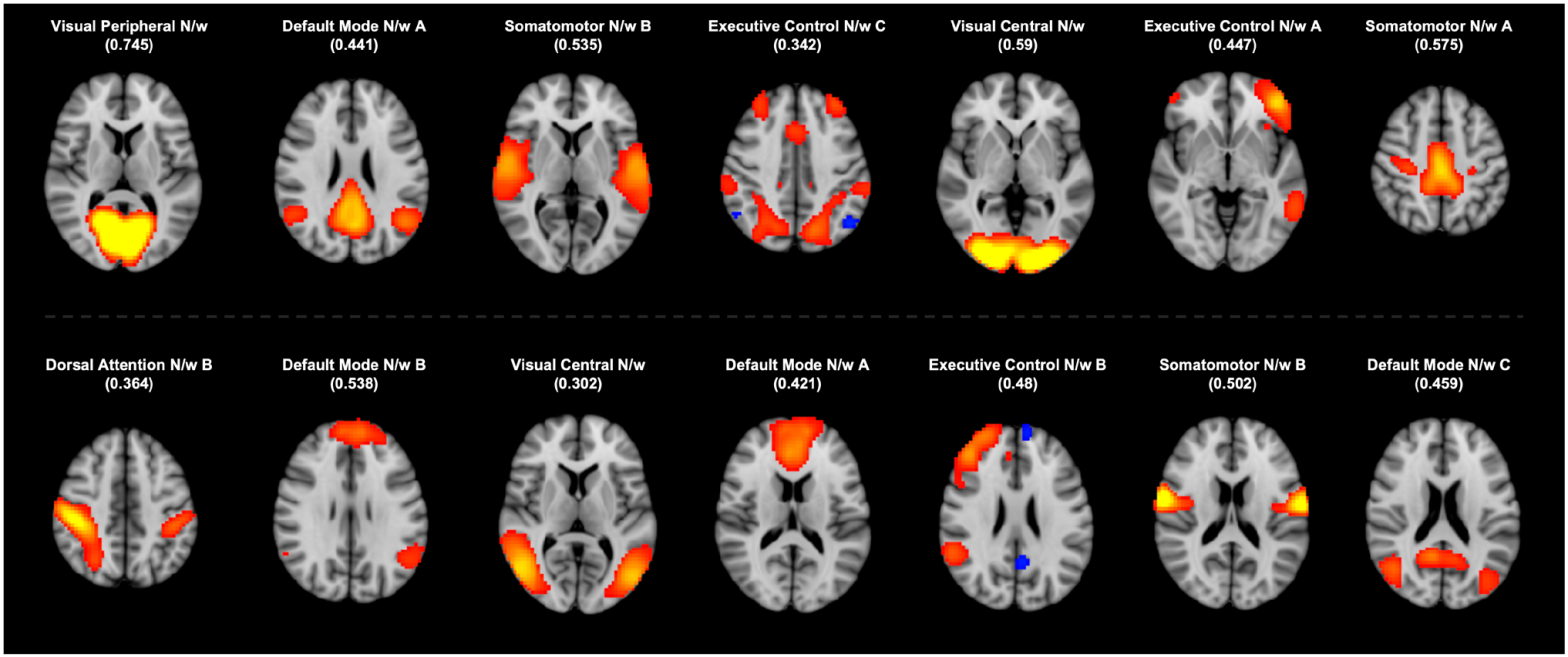
Fourteen resting-state networks (RSN) were identified by comparing the independent components (ICs) with network maps from the Yeo Atlas (https://surfer.nmr.mgh.harvard.edu/fswiki/CorticalParcellation_Yeo2011). The identified ICs are presented here with the best-matched RSN from the atlas. The values of corresponding correlation coefficients are provided in parentheses. Key: N/w = Network.

The BOLD time series of all subjects were concatenated in time and flattened into a 2D matrix. Group Independent Component Analysis (ICA) of this matrix was used to extract the predominant resting-state networks (RSNs) across all subjects. FSL’s MELODIC Tool (Calhoun et al., 2001) was used to implement this algorithm, extracting 20 independent components (ICs). Using AFNI’s 3dMatch tool, the ICs were classified into canonical RSNs based on their dice coefficient similarity with the respective networks in the Yeo Atlas (17-Networks) (Yeo et al., 2011). Among the 20 ICs, 14 ICs with dice coefficients greater than 0.254 were identified as canonical RSNs, which are shown in Figure 1. Dual regression (Filippini et al., 2009) was performed to obtain subject-wise time courses and spatial maps for each identified IC. The subject-specific maps of RSNs were compared across the cohorts to check for group-level differences.

#### 2.6.3 Statistical analysis

For each IC that was identified as a canonical RSN, we compared the subject-wise spatial maps of the network across the two cohorts using a permutation test. FSL’s randomize was used to conduct the test with 5000 permutations. Age and sex were added as covariates of no interest. Correction for multiple comparisons was done using the Threshold Free Cluster Enhancement (TFCE) method at p<0.05 (Smith & Nichols, 2009).

#### 2.6.4 Relationship with infection severity

The effect of infection severity on resting-state functional connectivity was studied using 47 HCs (32.30 ± 9.23 years, 11F), 14 HPs (41.21 ± 12.59 years, 5F), and 39 NHPs (27.49 ± 8.24 years, 7F). For each IC that was identified as a canonical RSN, we compared the subject-wise spatial maps of the three groups using permutation testing (N = 5000). Age and sex were added as covariates of no interest. Correction for multiple comparisons was performed using the TFCE method at p<0.05 . Using non-parametric permutation-based testing helped us mitigate potential bias from unequal group sizes in the HCs, NHPs, and HPs, as these tests are robust to unequal group sizes and variance heterogeneity.

### 2.7 Analysis of cerebral blood flow

Blood perfusion in the brain can be detected by using the arterial spin labeling (ASL) MRI technique. Here, a contrast is generated in the blood vessels of the brain by magnetically tagging incoming blood with an inverted spin (Detre et al., 1994). For the analysis of cerebral blood flow (CBF) using ASL images, we included 67 CRPs (32.21 ± 11.82 years, 15F) and 47 HCs (31.98 ± 8.95 years, 11F), excluding 9 CRPs and 4 HCs due to poor image quality.

#### 2.7.1 Data processing

With the ASL protocol described in earlier sections, difference images (tagged–control) were obtained. The data were processed using the Oxford ASL pipeline in FSL toolbox (Chappell et al., 2023). Preprocessing involved motion correction and spatial regularization. Anatomical images processed using the FSL-ANAT pipeline (Smith, 2002) were utilized to co-register the pre-processed difference images to native space, followed by normalization to MNI space using FSL. The obtained CBF values were calibrated using the M0 image to get the CBF in absolute units. Partial volume correction was then done to obtain separate perfusion maps for gray and white matter. These perfusion maps were used to perform a region analysis based on the ROIs defined in the Harvard–Oxford Atlas. Therefore, perfusion values for 68 ROIs were obtained and used for statistical comparison.

#### 2.7.2 Statistical analysis

Perfusion values were obtained for 68 ROIs. For each ROI, these values were compared across CRPs and HCs using the Mann–Whitney U Test. The results were corrected for multiple comparisons using the FDR-BH method at $p_{FDR}<0.05$ .

#### 2.7.3 Relationship with infection severity

CRPs were classified into NHPs and HPs to study the effect of severity on cerebral blood flow. We included 47 HCs (31.98 ± 8.95 years, 11F), 42 NHPs (27.12 ± 7.84 years, 8F), and 17 HPs (40.23 ± 12.21 years, 4F) in this analysis. The comparison was done using the Kruskal–Wallis H-test. Multiple-comparison correction was performed using the FDR-BH method at $p_{FDR}<0.05$ .

## 3 Results

### 3.1 Subject demographics and characteristics

**Fig. 2.**
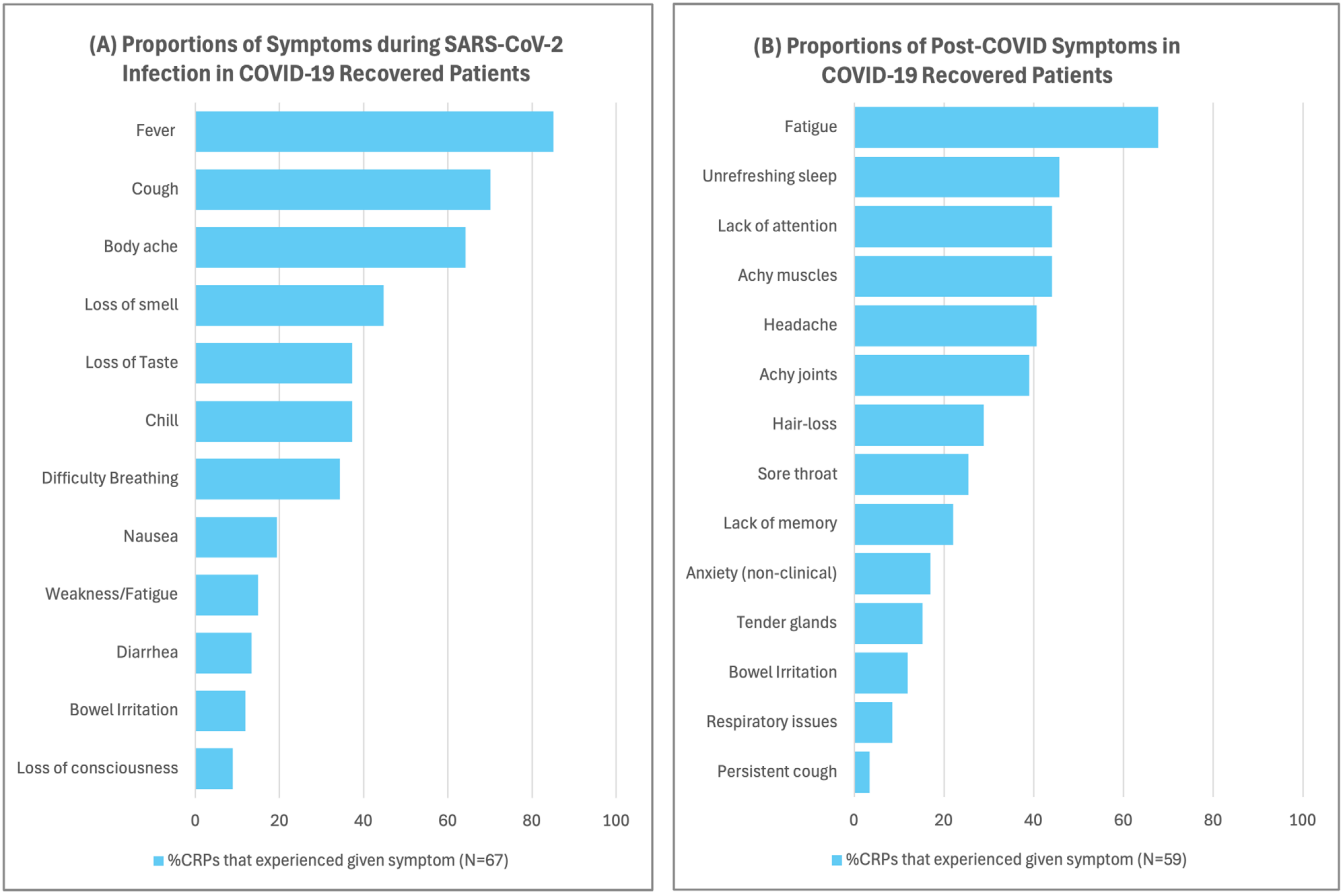
(A) Proportions of symptoms during SARS-CoV-2 infection in COVID-19 recovered patients, total CRPs = 67. (B) Proportions of post-COVID symptoms in COVID-19 recovered patients. We also requested CRPs to share their post-COVID experience and any persistent symptoms since recovery. Among 59 (30.85 ± 10.52 years, 15F) consenting CRPs, fatigue (40/59) was the most commonly reported post-COVID symptom, followed by unrefreshing sleep (27/59) and lack of attention (26/59). Other symptoms reported by CRPs were achy muscles (26/59), achy joints (23/59), and headache (24/59). A summary of the post-COVID symptoms is presented in (B).

For this study, we recruited a total of 127 subjects. The demographic details of the cohorts included in our analysis are presented in Table 1. In the CRP group, 67 subjects consented to sharing their treatment history. Based on treatment history, 46 CRPs were classified as NHPs. The other 21 patients were classified as HPs and had undergone hospitalization during their treatment, requiring oxygenation (10/21), administered remdesivir (10/21), or steroids (3/21). The most commonly experienced symptoms for the CRPs were fever (85.07%) and cough (70.15%), followed by body ache (64.17%), loss of smell (44.77%), chills (37.31%), loss of taste (37.31%), breathing difficulties (34.33%), nausea (19.40%), and weakness (14.92%). Figure 2A presents a summary of the range of symptoms that were experienced by the CRPs in our study.

**Table 1.** Demographic details of the participants recruited for this study.

	COVID-19 recovered participants			Healthy controls		
	Number	Age (years)	Sex	Number	Age (years)	Sex
Total recruited	76	32.38 ± 11.52	19 F/ 57M	51	31.98 ± 8.96	12 F/ 39M
Shared COVID-19infection details	67	31.12 ± 10.78	16 F/ 51M	NA		
Shared experience withpost-COVID symptoms	59	30.85 ± 10.52	15 F/ 44M	27	30.33 ± 7.85	6 F/ 21M

The age distribution is represented using the “mean ± standard deviation” values.

### 3.2 Differences between COVID-19 survivors and controls

#### 3.2.1 No difference in whole-brain and cortical morphology between cohorts

First, as a baseline, we performed a comparison of the whole-brain morphological features. We found no significant differences in TIV, BTV, GMV, WMV, CSFV, GMVnorm, WMVnorm, or CSFVnorm between CRPs and HCs ($p_{FDR}<0.05$ ). Further, we compared the morphological features of the cortical ROIs among the cohorts. The cortex was segmented into 68 ROIs based on the Desikan–Killiany atlas, and the volume and average thickness of the 68 ROIs, along with the mean thickness and volume of each hemisphere, were determined. Comparison of ROI volumes between the cohorts yielded no significant differences ($p_{FDR}<0.05$ ). Moreover, the thickness of the cortical ROIs also showed no significant differences when compared across the CRP and HC cohorts ($p_{FDR}<0.05$ ).

#### 3.2.2 Reduced ROI volume in right basal ganglia and limbic system

**Fig. 3.**
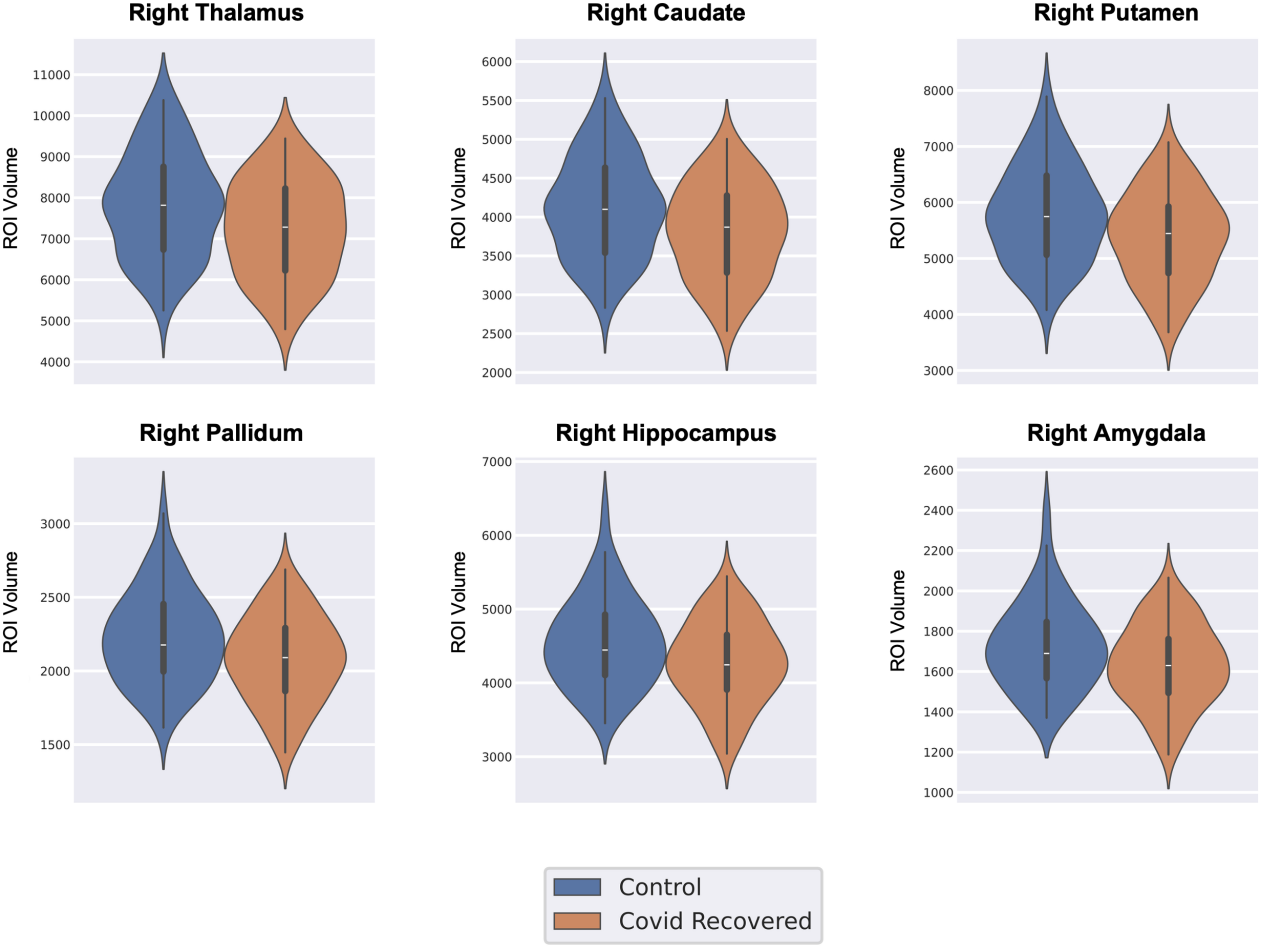
Distribution of volume of regions that showed significant differences between the COVID-19 recovered patients (CRPs) and healthy controls (HCs) ($p_{FDR}<0.05$ ).

The volumes of 15 subcortical ROIs were computed for each subject and normalized to the TIV. Statistical comparison of the subcortical ROI volumes indicated significant differences in six regions ($p_{FDR}<0.05$ ) in the right hemisphere. The regions identified were the thalamus, caudate, putamen, pallidum, hippocampus, and the amygdala. All the ROIs showed reduced volume in CRPs as compared with HCs. It was observed that the putamen, caudate nucleus, and pallidum are essential regions of the basal ganglia in the brain. Moreover, the thalamus, hippocampus, and amygdala are crucial components of the limbic system. Figure 3 shows the distribution of subcortical volumes for the ROIs that exhibited a significant difference between the cohorts.

#### 3.2.3 CRPs show reduced gray-matter volume

Using SBM, the thickness and volume of gray-matter tissue in the cortex were compared across HCs and CRPs over a reconstructed surface. A GLM was used to test the changes at each vertex of the surface. *p*-values were corrected for multiple comparisons using the FDR method.

**Fig. 4.**
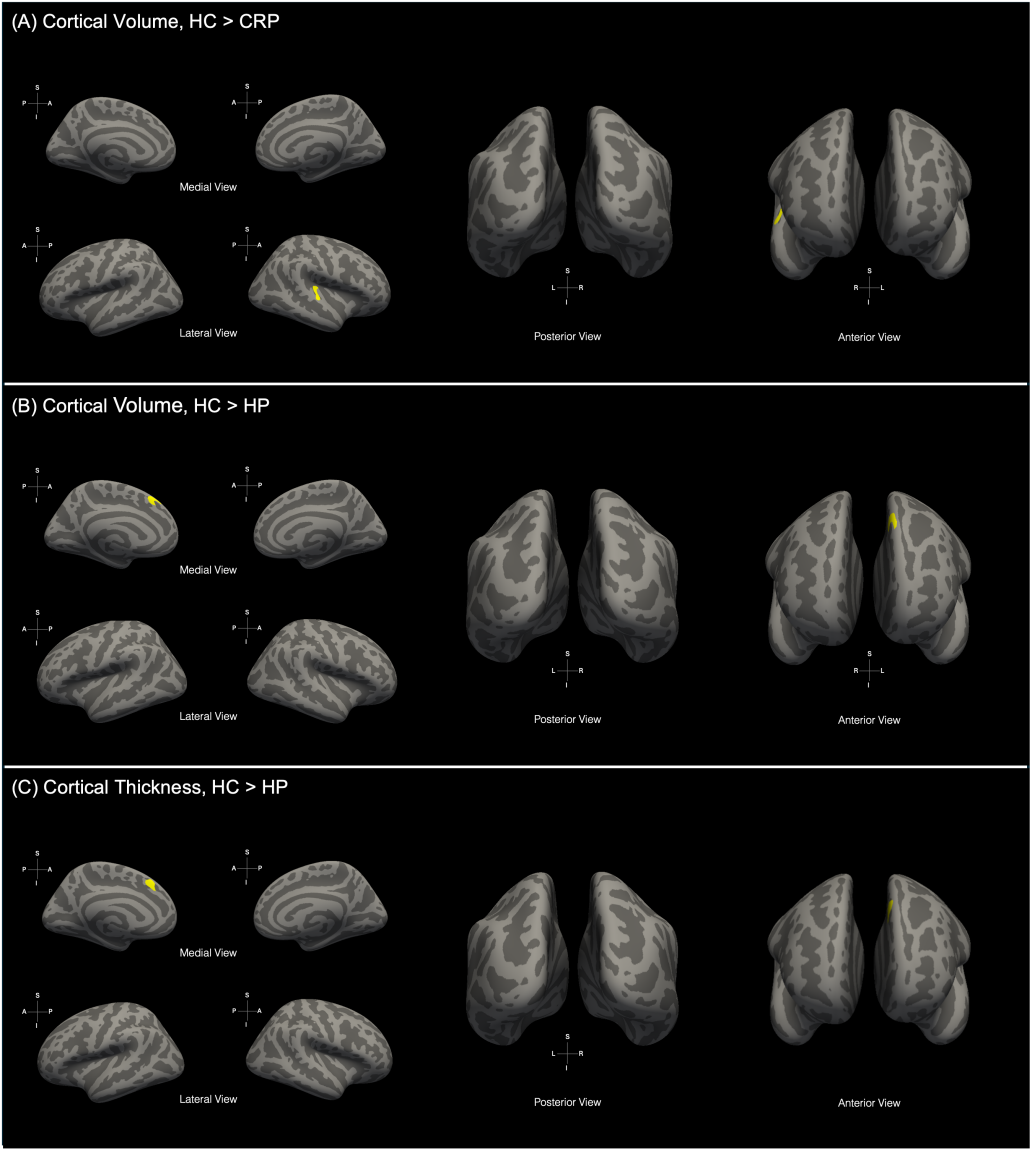
Significant clusters obtained upon comparison of surface-based morphological metrics: (A) Cortical volume differences between healthy controls (HCs) and COVID-19 recovered patients (CRPs) where cluster was observed in the right superior temporal gyrus, insular cortex ($p_{FDR}<0.05$ , HC>CRP). Significant differences in (B) cortical volume and (C) cortical thickness were observed between HCs and hospitalized patients (HP) wherein the cluster was found left medial prefrontal cortex ($p_{FDR}<0.05$ , HC>HP).

Comparison of cortical GM thickness between HCs and CRPs did not highlight any significant clusters ($p_{FDR}<0.05$ ). However, comparison of cortical GM volume across the cohorts revealed a significant cluster with reduced volume in CRPs ($p_{FDR}<0.05$ , HC>CRP). The cluster overlaps the posterior parts of the right superior temporal gyrus and covered parts of the insular cortex (Fig. 4A). Table 2 summarizes the details of the cluster reported here.

**Table 2.** Summary of clusters observed showing significant differences in cortical thickness and cortical volume among the healthy controls (HCs), COVID-19 recovered patients (CRPs), non-hospitalized patients (NHPs), and hospitalized patients (HPs).

Cortical thickness						
Contrast	Peak t-score	Size ($mm^{2}$)	Peak X	Peak Y	Peak Z	Region identified
HC>HP	3.4556	204	-7.2	22.9	43.8	Left medial prefrontal cortex

Cortical volume						
Contrast	Peak t-score	Size ($mm^{2}$)	Peak X	Peak Y	Peak Z	Region identified
HC>CRP	4.222	216.62	48.7	-24.1	4.8	Right superior temporal gyrus, insular cortex
HC>HP	3.7983	197.44	-6.7	24.7	42.9	Left medial prefrontal cortex

#### 3.2.4 Microstructural differences in tracts of the limbic system

The AFQ algorithm was employed to trace and identify 20 white-matter tracts in the brain for each subject. For each tract, four measures (FA, MD, AD, RD) were calculated that quantify the microstructural integrity of the tracts. These values were compared between the CRPs and HCs to test for any post-COVID differences in white-matter integrity.

**Fig. 5.**
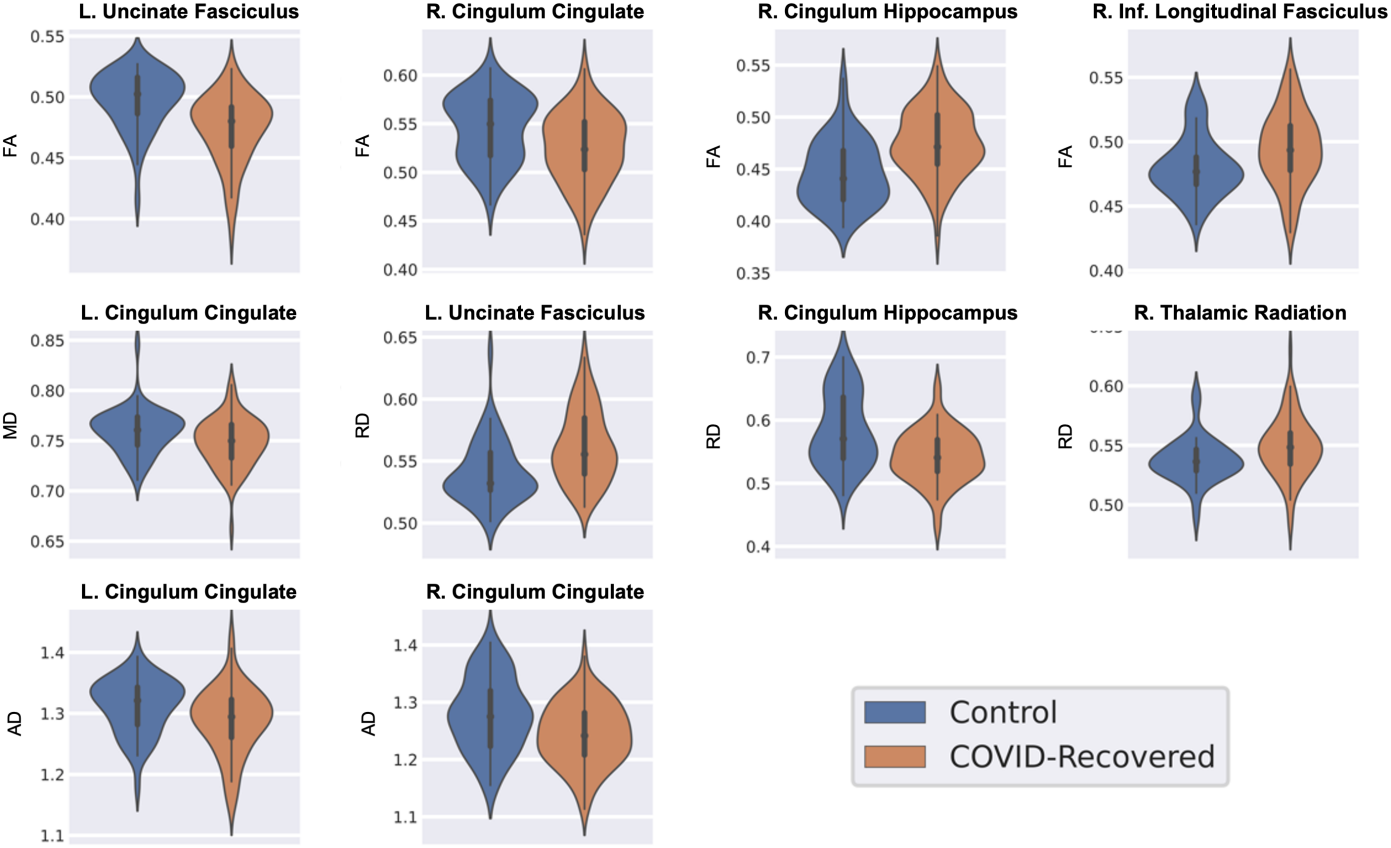
Distribution of diffusion metrics of tracts that showed a significant difference between healthy controls (HCs) and COVID-19 recovered patients (CRPs) ($p_{FDR}<0.05$ ). Key: L. = left, R. = right, FA = fractional anisotropy, MD = mean diffusivity, AD = axial diffusivity, RD = radial diffusivity.

We found four tracts that exhibited significant alterations in FA among the groups ($p_{FDR}<0.05$ ). These results are consistent with the observations on the subset of these data, as reported in Mishra et al. (2024). The left UF and the right CC showed reduced values of FA in the CRPs compared with HCs. However, the right CH and the right ILF had greater FA values in CRPs. Further, significant aberrations in MD were observed in three white-matter tracts, including the left CC, left UF, and right CH ($p_{FDR}<0.05$ ). The left UF exhibited higher MD in the CRPs as compared with HCs, while MD was lower in the CRPs for left CC and right CH. The comparison also highlighted significant alterations in RD for left UF, right CH, and right ATR ($p_{FDR}<0.05$ ). RD was elevated in the CRPs as compared with HCs for the left UF and right ATR, while it showed a decrease for the right CH. Furthermore, two tracts also showed significant differences in AD values between the two cohorts ($p_{FDR}<0.05$ ). We found that the left and right CC had reduced AD in the CRPs as compared with the HCs. Figure 5 shows the violin plots of the distribution of diffusion measures described above.

#### 3.2.5 No group differences in activity of resting-state networks

FSL’s MELODIC tool was used to extract 20 ICs across all subjects. Among these, 14 ICs were identified as canonical RSNs using dice coefficients. ICs with high spatial overlap with RSNs from the Yeo Atlas were labeled and included in the statistical comparison. The identified ICs are shown in Figure 1 along with respective dice coefficients. For each identified IC, we compared the subject-wise spatial maps across the two cohorts using a permutation test. The test revealed no significant cluster with *p* < 0.05 in any of the RSNs.

#### 3.2.6 No significant group differences in cerebral blood flow

We compared the average blood perfusion values for 68 ROIs across HCs and CRPs. We did not find significant differences in the perfusion values in any of the ROIs at $p_{FDR}<0.05$ . The ROI that showed the strongest contrast between the cohorts was the left pallidum with $p_{unc}=0.0926$ .

### 3.3 Effect of severity of COVID-19 infection

Next, we investigated whether these brain changes were associated with the severity of the COVID-19 infection. Based on available treatment history of 67 CRPs, 46 CRPs were classified as NHPs. The other 21 patients who had undergone hospitalization during their treatment (requiring oxygenation, administered remdesivir, or steroids) were classified as HPs.

#### 3.3.1 Whole-brain morphology not linked to severity

The Kruskal–Wallis H-test detected no significant effect of severity within HC, NHP, and HP groups for whole brain features: TIV, BTV, GMV, WMV, CSFV, GMVnorm, WMVnorm, or CSFVnorm ($p_{FDR}<0.05$ ). As no significant changes linked to severity were observed, post hoc tests were not performed.

#### 3.3.2 ROI-level morphological changes linked to infection severity

The Kruskal–Wallis H-test was used to test the effect of infection severity on the cortical volume and thickness across 70 cortical ROIs. No significant effect of severity was observed in the case of the cortical ROIs defined using the Desikan–Killiany atlas ($p_{FDR}<0.05$ ).

**Fig. 6.**
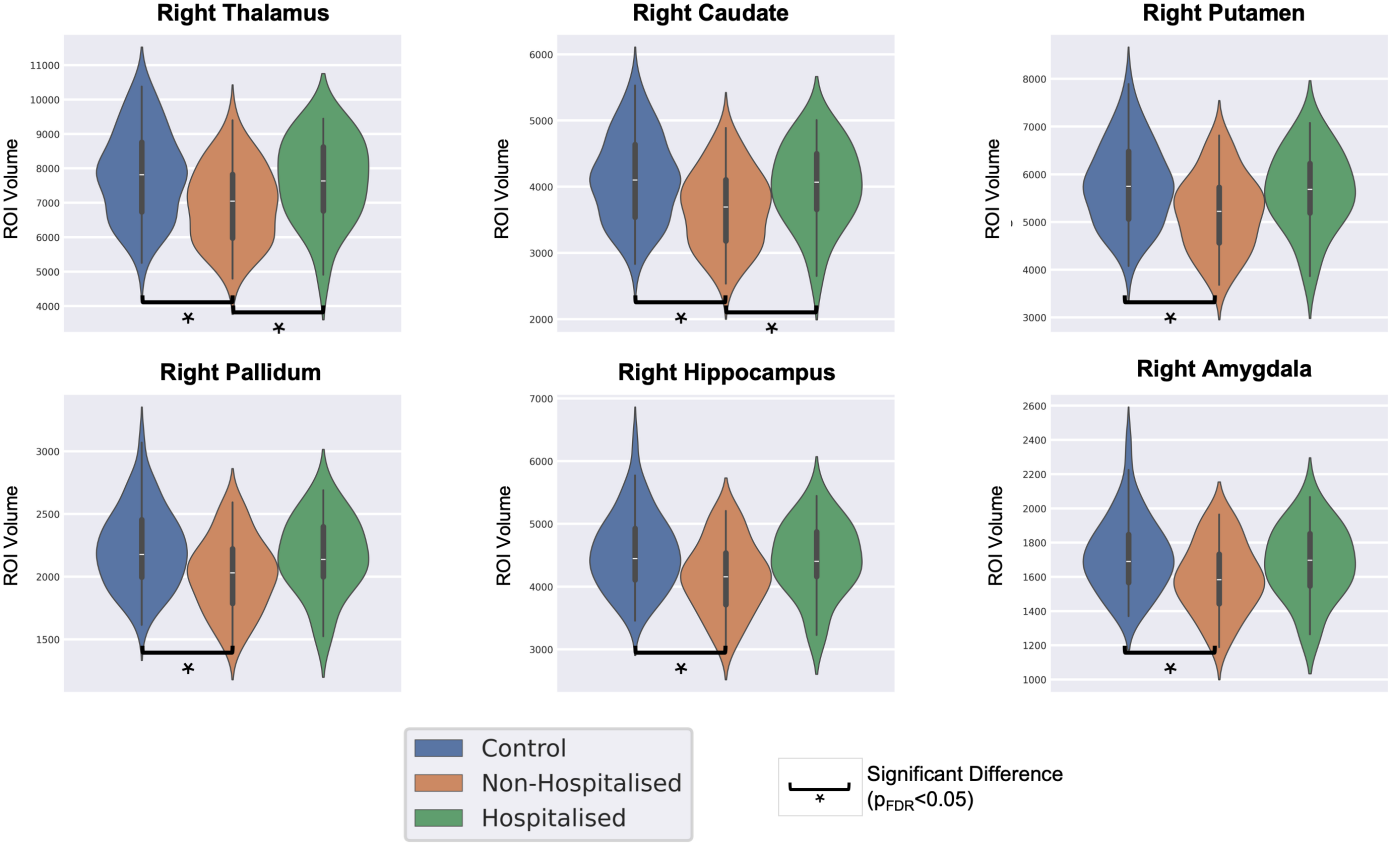
Distribution of volume of regions that showed a significant effect of infection severity among healthy controls (HCs), non-hospitalized patients (NHPs), and hospitalized patients (HPs) ($p_{FDR}<0.05$ ).

However, a significant effect of infection severity on the subcortical ROIs ($p_{FDR}<0.05$ ) was observed. The effect of severity on sub-cortical volume was significant in the right thalamus, right caudate, right putamen, right pallidum, right amygdala, and the right hippocampus ($p_{FDR}<0.05$ ). Post hoc Dunn’s tests were conducted for these ROIs. For all six ROIs tested, it was noted that the NHPs had significantly reduced volume as compared with the HCs ($p_{FDR}<0.05$ ), while no significant differences were observed upon comparing HCs and HPs. Additionally, in the case of the thalamus and caudate, the NHPs had significantly less volume as compared with the HPs. Figure 6 shows the violin plots of the distribution of subcortical volumes for the ROIs that exhibited a significant effect of infection severity.

#### 3.3.3 Gray-matter thickness and volume linked with infection severity

The effect of infection severity on GM thickness and volume was tested using a GLM. Using an F-test, we observed a significant effect of severity on cortical volume in HCs, NHPs, and HPs ($p_{FDR}<0.05$ , $p_{unc}<0.05$ ). Post hoc and pair-wise tests were conducted to identify the groups with significant differences.

Pair-wise tests on the cortical GM thickness highlighted significant differences between the HC and HP groups. Comparison between these groups yielded a significant cluster in the left medial prefrontal cortex ($p_{FDR}<0.05$ , $p_{unc}<0.001$ , Fig. 4B). Post hoc comparisons of cortical GM volume also highlighted significant differences. Comparison of HCs with HPs revealed a significant cluster in the left medial prefrontal cortex ($p_{FDR}<0.05$ , $p_{unc}<0.001$ , Fig. 4C). The details of the clusters are presented in Table 2.

#### 3.3.4 Microstructural differences between HCs, NHPs, and HPs

**Fig. 7.**
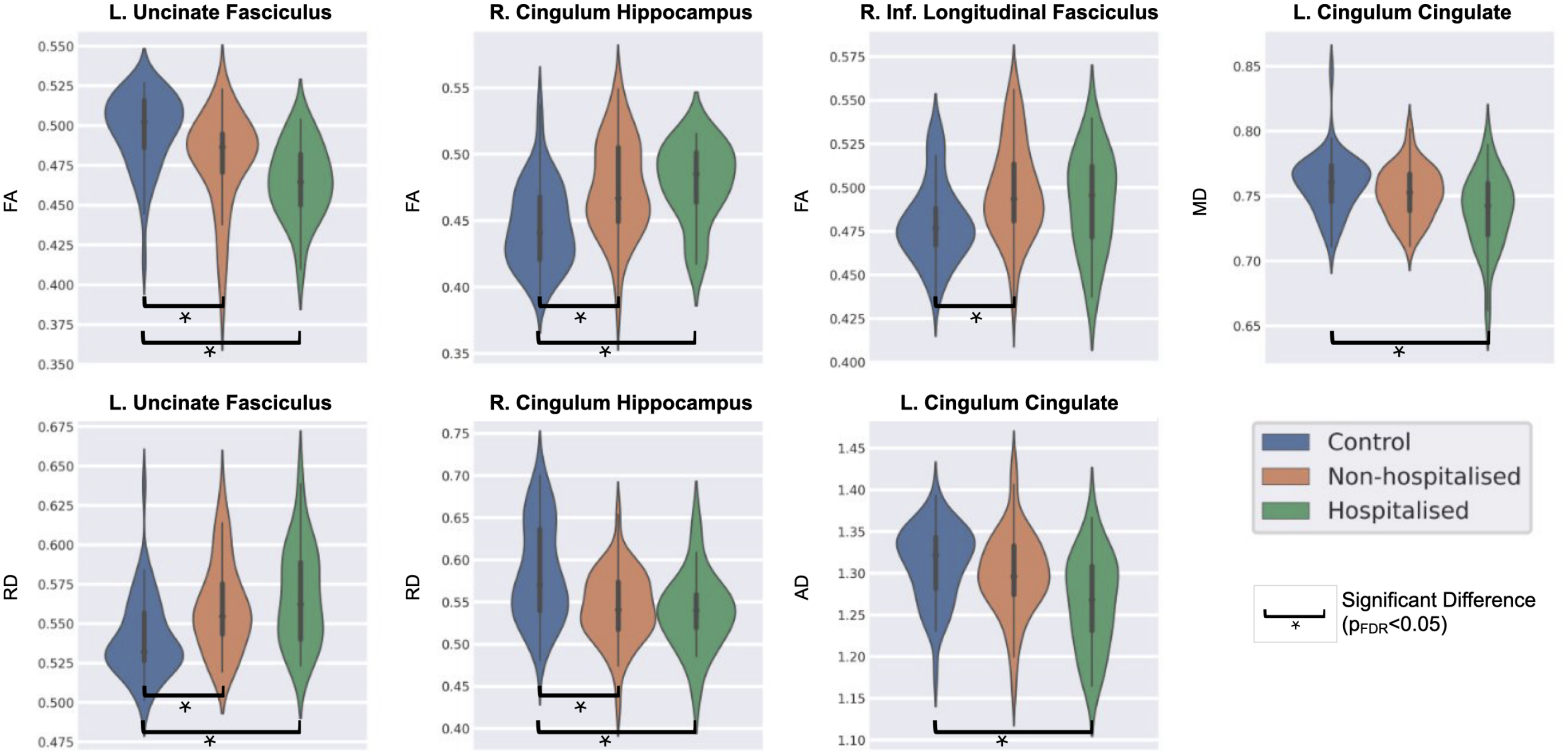
Distribution of diffusion metrics of tracts that showed a significant effect of infection severity among healthy controls (HCs), non-hospitalized patients (NHPs), and hospitalized patients (HPs) ($p_{FDR}<0.05$ ). Key: L. = left, R. = right, FA = fractional anisotropy, MD = mean diffusivity, AD = axial diffusivity, RD = radial diffusivity, FDR = false detection rate.

The Kruskal–Wallis H-test detected significant differences between HC, NHP, and HP for four tracts ($p_{FDR}<0.05$ ). The left UF and right CH showed a significant effect of severity in FA and RD; the left CC showed significant changes linked to severity in MD and AD, and the right ILF exhibited a significant effect of infection severity on FA. Figure 7 presents the violin plots of the distribution of diffusion measures across the HCs, NHPs, and HPs for the results described above.

Further, upon conducting post hoc tests on these measures for pairwise comparison between the groups, we found that none of the tracts’ diffusion measures showed a significant difference between the HP and NHP cohorts (p<0.05 ). Significant pairwise alterations (p<0.05 ) were, however, observed between the HC and NHP groups in the left UF (FA and RD), right CH (FA and RD), and the right ILF (FA). The left CC did not show significant differences between HC and NHP groups for p<0.05 . Additionally, pairwise comparison of HC and HP groups highlighted significant differences (p<0.05 ) in the left UF (FA and RD), right CH (FA and RD), and the left CC (MD and AD). We did not find any significant differences between the HC and HP groups in the right ILF at a p<0.05 threshold.

#### 3.3.5 Hospitalized patients show significant functional changes

Upon testing each of the 14 identified RSNs, we observed a significant effect of infection severity in four RSNs ($p_{corr}<0.05$ ), namely, default mode network—A (DMN-A), DMN-C, somatomotor network—A (SMN-A), and the somatomotor network—B. Further, we conducted post hoc pairwise comparisons for these networks between HCs, NHPs, and HPs.

**Fig. 8.**
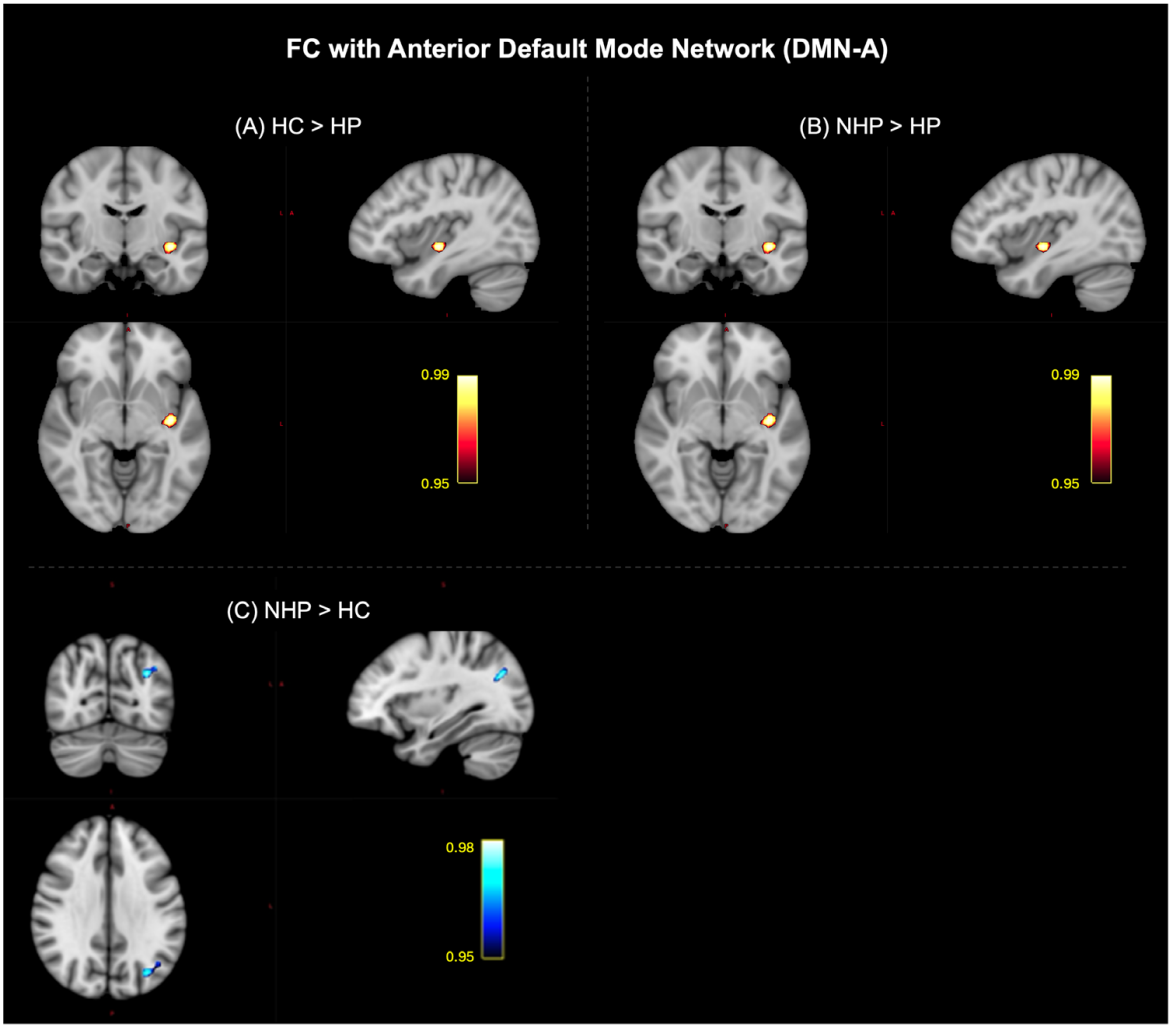
Significant clusters that highlight the effect of infection severity on the functional connectivity (FC) of the anterior default mode network (DMN-A) with the brain among healthy controls (HCs), Non-hospitalized patients (NHPs), and hospitalized patients (HPs) ($p_{corr}<0.05$ ). (A) Post hoc comparison of HCs and HPs showed a significant cluster in the left posterior insular cortex (HC>HP). (B) Post hoc comparison of HPs and NHPs also highlighted a cluster in the left posterior insular cortex (NHP>HP). (C) Post hoc comparison of NHPs and HCs showed a significant cluster in the dorsal visual association cortex (NHP>HC).

**Fig. 9.**
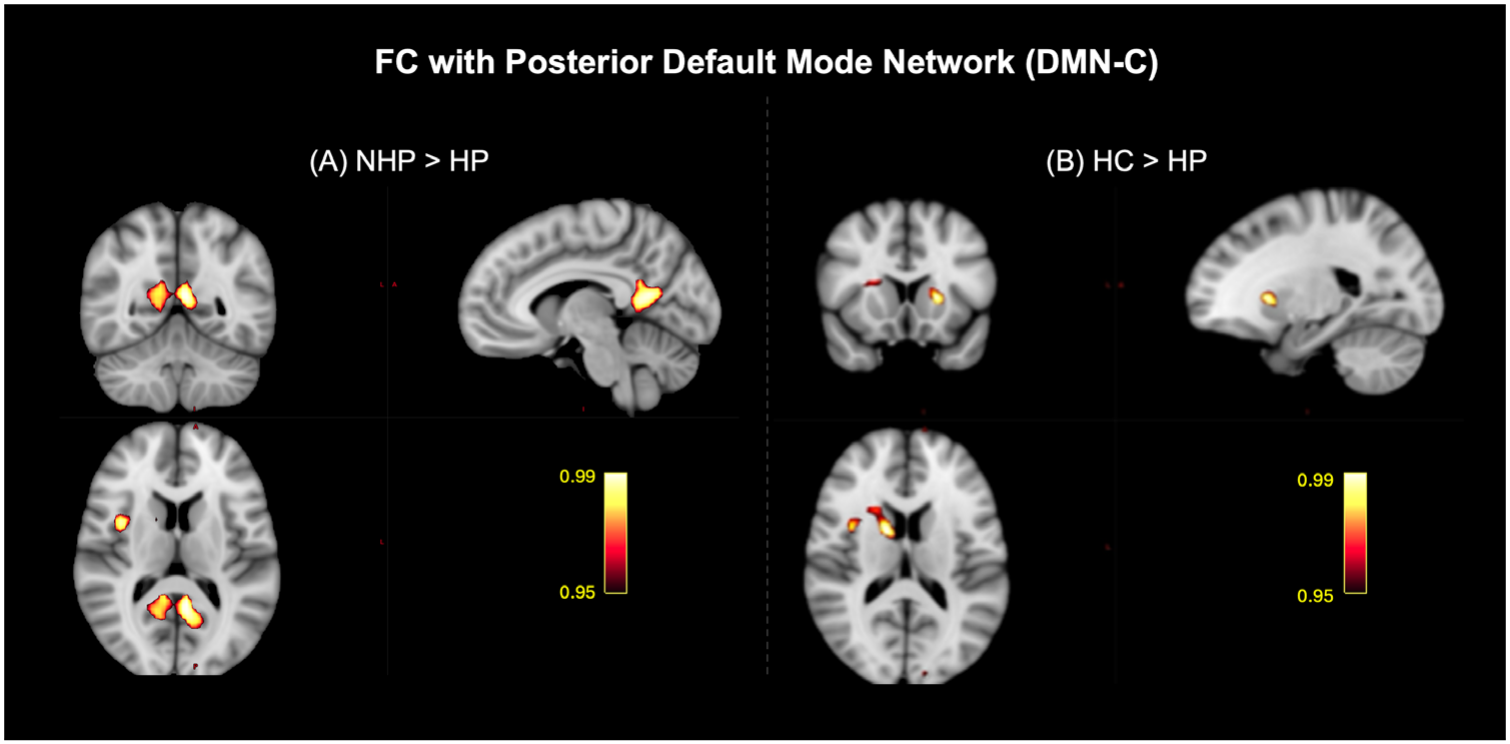
Significant clusters that highlight the effect of infection severity on the functional connectivity (FC) of the posterior default mode network (DMN-C) with the brain among healthy controls (HCs), non-hospitalized patients (NHPs), and hospitalized patients (HPs) ($p_{corr}<0.05$ ). (A) Post hoc comparison of NHPs and HPs highlighted clusters in the precuneus, posterior cingulate, and the inferior frontal cortex (NHP>HP), and (B) post hoc comparison of HCs and HPs showed a significant cluster in the right caudate and the left thalamus (HC>HP).

The spatial maps of the DMN-A showed significant differences between the NHPs and HPs ($p_{corr}<0.05$ , NHP > HP) in the left posterior insular cortex, which can be observed in Figure 8. Significant clusters were also obtained upon comparing HCs and HPs ($p_{corr}<0.05$ , HC > HP) in the same region as shown in Figure 8. Additionally, comparing the HCs and NHPs showed significant differences in the dorsal visual association cortex ($p_{corr}<0.05$ , NHP > HC) as shown in Figure 8. Comparison of the spatial maps of the DMN-C between the NHPs and HPs highlighted significant differences in the precuneus, posterior cingulate, and the inferior frontal cortex ($p_{corr}<0.05$ , NHP > HP) as shown in Figure 9. Further, significant differences in FC with DMN-C between HCs and HPs were observed in the right caudate and the left thalamus ($p_{corr}<0.05$ , HC > HP) as shown in Figure 9.

**Fig. 10.**
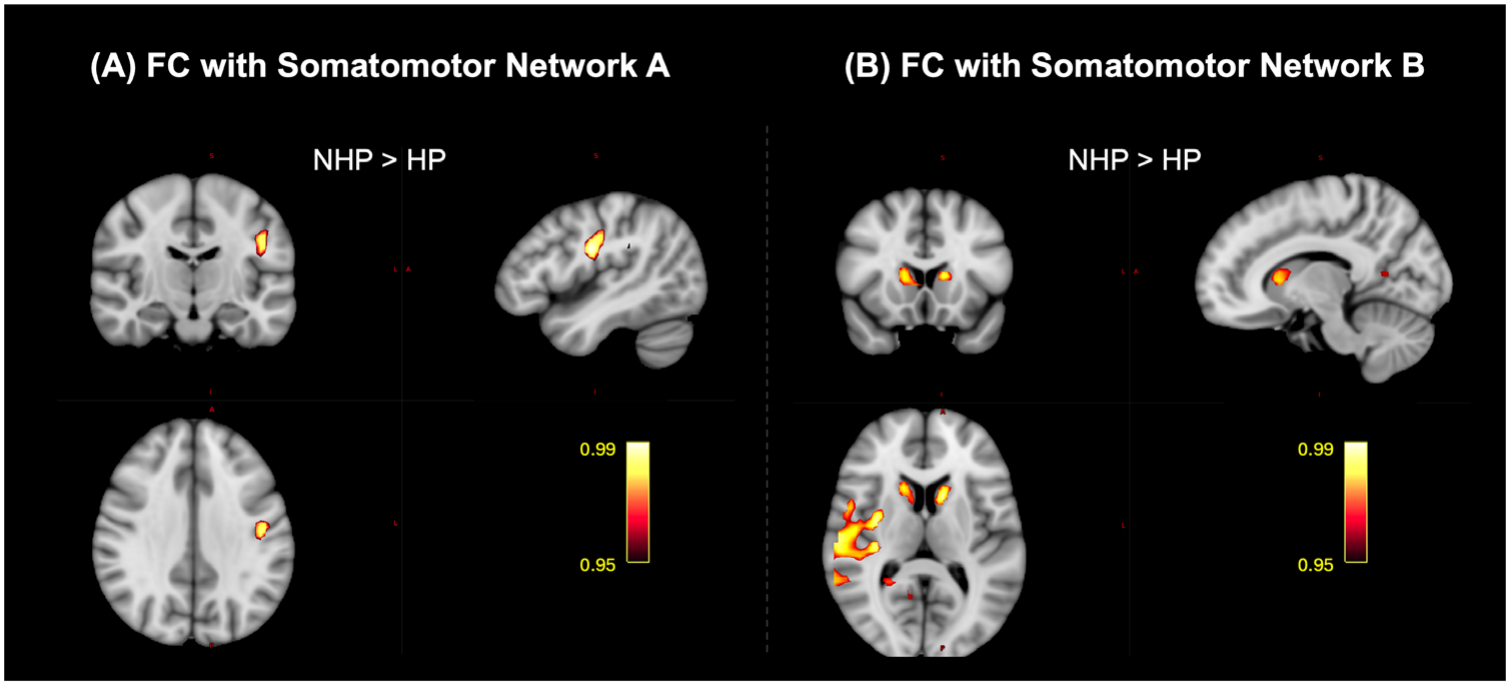
Significant clusters that highlight the effect of infection severity on the functional connectivity (FC) of the somatomotor network (SMN) with the brain among healthy controls (HCs), non-hospitalized patients (NHPs), and hospitalized patients (HPs) ($p_{corr}<0.05$ ). (A) Post hoc comparison of the FC of the SMN-A in the NHPs and HPs highlighted a significant cluster in the left postcentral gyrus (NHP>HP) and (B) post hoc comparison of the FC of the SMN-B in the NHPs and HPs highlighted a significant cluster in the left and right caudate, the right Heschl’s and supramarginal gyri (NHP>HP).

Spatial maps of the Somatomotor-A network showed significant differences between the NHPs and HPs in the left postcentral gyrus ($p_{corr}<0.05$ , NHP > HP, Fig. 10). FC with the Somatomotor-B network exhibited significant differences between the NHPs and HPs in the left and right caudate, the right Heschl’s gyrus, and right supramarginal gyrus ($p_{corr}<0.05$ , NHP > HP) as shown in Figure 10. For both networks, the comparison of HCs with NHPs and HCs with HPs did not highlight any significant differences ($p_{corr}<0.05$ ). A summary of the clusters obtained is provided in Table 3.

**Table 3.** Summary of clusters observed showing significant differences in resting-state functional connectivity among the healthy controls (HCs), COVID-19 recovered patients (CRPs), non-hospitalized patients (NHPs), and hospitalized patients (HPs).

Resting-state network	Contrast	Peak confidence (1-p)	Size (voxels)	Peak X	Peak Y	Peak Z	Region identified
Default mode network-A	HC >HP	0.998	38	-36	-12	-6	Left insular cortex
	NHP>HP	0.999	100	-36	-12	-9	Left insular cortex
		0.964	21	3	-18	48	Cingulate gyrus, posterior division
	NHP >HC	0.989	49	-33	-69	30	Lateral occipital cortex, superior division
Default mode network-C	HC >HP	0.991	87	15	3	15	Right caudate, central opercular cortex
		0.991	32	-21	15	3	Left putamen
	NHP >HP	0.995	263	-6	-51	12	Precuneus cortex
		0.992	56	39	3	12	Inferior frontal cortex
Somatomotor network-A	NHP >HP	0.995	77	-45	-9	27	Postcentral gyrus
Somatomotor network-B	NHP >HP	0.994	927	63	-42	18	Central opercular cortex, Heschl’s gyrus
		0.992	164	-12	12	9	Bilateral caudate
		0.984	90	54	-33	51	Supramarginal gyrus, anterior division

## 4 Discussion

In this multimodal MRI study of long-term neurological sequelae among COVID-19 survivors, we leveraged structural, diffusion, functional, and perfusion imaging to study alterations in the brain. By comparing 76 CRPs with 51 HCs, and further classifying CRPs into hospitalized (HP; N = 21) and non-hospitalized (NHP; N = 46) subgroups, we delineated neurological effects in CRPs and how infection severity modulates microstructural and functional connectivity. Below, we integrate our findings with the existing literature, explore plausible mechanistic underpinnings, acknowledge study limitations, and outline future directions.

### 4.1 Regional specificity of structural changes

We conducted a detailed analysis of the morphological changes in the brain as a long-term effect of the COVID-19 infection. We started by comparing TIV, GMV, and WMV between the HCs and CRPs to test for any signs of widespread cortical atrophy. We observed that the TIV, GMV, and WMV did not differ between CRPs and HCs. Instead, subcortical analysis revealed selective volume reductions in right-hemisphere basal ganglia (putamen, caudate, pallidum) and limbic nodes (thalamus, hippocampus, amygdala). Our findings align with Hiene et al. (2023) who similarly observed basal ganglia atrophy in the COVID-19 recovered patients. Structural damage in the limbic system has also been consistently observed in COVID-19 survivors, ranging from GM loss in the thalamus (Jin et al., 2024) to changes in the orbitofrontal cortex (Paolini et al., 2023), hippocampal and parahippocampal regions (Dacosta-Aguayo et al., 2024; Dıez-Cirarda et al., 2023; Paolini et al., 2023). The absence of global changes alongside focal subcortical loss suggests that SARS-CoV-2 may preferentially target deep gray nuclei, perhaps via microvascular injury or direct neurotropism, rather than inducing diffuse neurodegeneration. Surface-based morphometry further localized cortical thinning and volume loss to paralimbic regions: the medial prefrontal cortex, insula, and posterior superior temporal gyrus. These loci have been implicated in attentional control, salience detection, and reward processing; functions commonly reported as impaired in “brain fog” and fatigue (lack of motivation) (Badenoch et al., 2022; Uddin, 2016). Our results extend prior studies (Arrigoni et al., 2024; Niu et al., 2025) by demonstrating that even subtle cortical alterations are reproducible across cohorts and imaging platforms.

Research on brain alterations following COVID-19 has produced mixed results. While some studies report widespread changes (Hosp et al., 2024; Perlaki et al., 2024), others have described a more localized effects or even increases in gray-matter volume in specific regions (Dacosta-Aguayo et al., 2024; Dıez-Cirarda et al., 2023; Douaud et al., 2022; Heine et al., 2023; Jin et al., 2024; Paolini et al., 2023). Within this context, our finding of no global brain volume loss adds nuance. Our results suggest that the neurological impact of COVID-19 may be more targeted, affecting specific neural circuits rather than producing uniform, widespread atrophy. Because our CRP cohort was scanned within 3–6 months of recovery, it remains possible that the observed atrophy represents an early stage of broader degeneration. However, this seems unlikely, as recent longitudinal studies—including CRPs scanned up to 2 years after recovery (Pacheco-Jaime et al., 2025)—continue to report localized morphometric changes without evidence of widespread gray-matter abnormalities.

### 4.2 Microstructural integrity of limbic white matter

The dMRI analysis revealed that UF exhibited decreased FA and increased RD in CRPs relative to HCs, with greater deviations in the HP subgroup. These changes are consistent with axonal injury and edema in these regions. The UF interconnects orbitofrontal and anterior temporal regions, supporting emotional regulation and memory retrieval (Rolls, 2004; Von Der Heide et al., 2013). Concurrent abnormalities in cingulum bundle subdivisions (CC, CH) further suggest a systemic disruption of limbic circuitry. These results are the extension of the outcomes reported in our previous study (Mishra et al., 2024). These patterns of damage to limbic white matter were also observed by Dıez-Cirarda et al. (2023), Paolini et al. (2023), and Kremer et al. (2020), who reported elevated MD and altered AD in the cingulum of post-COVID individuals. The combination of increased FA and lowered RD is consistent with extracellular fluid accumulation (edema), potentially reflecting stressed intra-axonal space or a microvascular leak in this tract. While the literature supports the role of inflammatory pathways in post-COVID neurological symptoms (Dos Reis et al., 2024; Vanderheiden & Klein, 2022), our findings could also be explained by alternative or overlapping processes such as microvascular injury, demyelination, or secondary neurodegeneration. Moreover, the interpretation of DTI metrics is often imprecise and susceptible to pitfalls (Figley et al., 2022). It is, therefore, difficult to conclusively identify the nature of microstructural damage using DWI without specific tests with PET ligands or cytokine markers. Nonetheless, despite these limitations, our DWI results are robust and consistently point to system-wide alterations in connectivity, particularly within the limbic system.

### 4.3 Functional connectivity alterations with severity

Resting-state ICA did not detect cohort-wide network differences, but stratification by severity uncovered that HPs exhibited significantly reduced connectivity between the default mode network (DMN-A) and insular cortex, as well as between DMN components and the caudate nucleus. The insula–DMN coupling underpins the ability to flexibly shift between internally and externally focused attention (Tegelbeckers et al., 2015); its disruption may, therefore, underlie deficits in sustained attention and cognitive flexibility. Reduced DMN–caudate connectivity also correlates with mood dysregulation, anhedonia in depression (Bluhm et al., 2009), and sleep disturbances (Jones & Ikuta, 2022), offering a potential link to the lack of motivation, insomnia, and fatigue reported by many survivors. Our findings build on Leitner et al. (2024) and Voruz et al. (2023) by showing that the severity of the acute infection predicts enduring functional network impairments. However, it is important to note that the mean age of HPs was higher than NHPs or HCs in our cohort. Even though we used age as a covariate of no interest in our statistical model, it is possible that age-related decline in RSFC could be a contributing factor in the reduced RSFC observed in HPs (Malagurski et al., 2022).

It should also be noted that we did not see the effect of COVID-19 infection when comparing HC with CRP. These effects were only highlighted upon stratifying CRPs based on infection severity. Specifically, HPs showed reduced RSFC as compared with both HCs and NHPs. This indicates that despite the strongly outlined anatomical and microstructural changes in both NHPs and HPs, the functional aberrations are either minimal in NHPs, or they are subtle and not easily detectable in the resting state. This suggests that the functional damage may not be a direct effect of the infection but instead could have developed slowly as a result of underlying tissue damage. Moreover, the functional deficits in NHPs may be sub-threshold in the resting state but contribute to behaviorally experienced cognitive deficits in task-based scenarios requiring cognitive effort and concentration. For instance, Madden et al. (2025) found that, while the DMN connectivity was retained, the RSFC correlated with neurocognitive performance in non-hospitalized CRPs with cognitive complaints. These findings shed valuable light on the contrast in the development of post-COVID pathophysiology in NHPs and HPs, exposing a link between infection severity and RSFC alterations.

### 4.4 Perfusion imaging and vascular changes

In contrast to previous reports of CBF reductions in post-COVID cohorts (Ajčević et al., 2023; Kim et al., 2023; Qin et al., 2021), our ASL analysis across 68 ROIs found no statistically significant group or severity effects after FDR correction. The strongest sub-threshold trend in the left pallidum may warrant targeted follow-up, but overall, these results suggest that macroscopic perfusion deficits, if present, may be transient or heterogeneous across individuals. However, a decrease in CBF has previously been reported in the thalamus, orbitofrontal cortex, and nodes of the basal ganglia (Kim et al., 2023). Another study reported a significant loss of CBF in the posterior cingulate cortex, superior medial frontal gyrus, and the insula (Qin et al., 2021). It is important to note that there are multiple factors that could lead to diverse findings across studies using ASL. In the aforementioned studies, the comparison of CBF was done using a voxel-wise method, which could be more sensitive to localized alterations. Another important factor that could influence observations is the reference used to quantify CBF values. Alternatively, our cross-sectional study, averaged at 3–6 months post-infection, may have missed early perfusion abnormalities that normalize over time.

Notably, while other studies reporting perfusion abnormalities in CRPs are uni-modal, focusing on ASL MRI, our study helps contextualize the (lack of) perfusion changes with respect to alterations in morphological, microstructural, and functional changes. Moreover, as the reports of loss in CBF in CRPs are less frequent than studies reporting structural, microstructural, or functional damage, it is likely that primary mode of damage by the coronavirus may not be vascular, that is, the abnormalities observed in CRPs are not directly linked to secondary complications in respiratory or circulatory systems in co-morbid patients. Instead, the pathophysiological mechanism of the post-COVID syndrome appears to be mediated by gray- and white-matter damage and subsequent functional disruption.

### 4.5 Mechanistic implications

The multimodal design of this study provides a comprehensive view of post-COVID syndrome, capturing morphological, microstructural, functional, and vascular aberrations. Morphological alterations in CRPs were not global, thereby ruling out widespread gray-matter atrophy. Instead, they were localized to subcortical regions of the limbic system and basal ganglia. White-matter changes were also detected in the uncinate fasciculus and cingulum bundle, whereas no group-level functional disruptions were evident when comparing CRPs with HCs. Stratifying CRPs by infection severity yielded additional insights: (a) both non-hospitalized (NHPs) and hospitalized patients (HPs) exhibited subcortical volumetric and microstructural alterations, (b) both groups showed cortical thinning in paralimbic regions, and (c) only HPs demonstrated functional connectivity disruptions relative to HCs and NHPs.

These findings can be interpreted within alternative models of post-COVID neuropathophysiology. One possibility is that gray- and white-matter damage reflects direct SARS-CoV-2 neurotropism, occurring independently of infection severity, whereas functional disruptions arise secondarily from severity-related systemic complications (e.g., respiratory or circulatory dysfunction). If this were the case, vascular abnormalities would be expected primarily in HPs, yet we did not detect such differences. An alternative model appears more consistent with our data: SARS-CoV-2 neurotropism may initially affect subcortical nuclei of the limbic system and basal ganglia and subsequently extend to paralimbic cortical regions via cortico-limbic tracts. Severity may modulate the extent, rather than the presence, of damage, as suggested by trends in gray- and white-matter alterations across severity levels (Figs. 6, 7), with functional disruptions emerging in the most severely affected patients. This framework also accounts for the absence of significant vascular alterations in both NHPs and HPs.

Therefore, collectively our findings support a model in which SARS-CoV-2 induces focal neuronal and axonal injury within deep gray nuclei and limbic networks, likely mediated by endothelial dysfunction, inflammatory cytokines, and possible neuroinvasion (Awogbindin et al., 2021). We found localized atrophy in basal ganglia and paralimbic cortices, compromised integrity of the uncinate fasciculus and cingulum bundle, and severity-dependent DMN decoupling in COVID survivors. Altogether, these imaging alterations correspond closely to their self-reported symptoms. A majority of CRPs (40/59) reported persistent fatigue, and 26 of 59 noted attentional lapses weeks after recovery. The focal damage to the deep gray nuclei of the basal ganglia and the insular cortex outlines abnormalities in the reward processing circuitry in CRPs, aligning with reports of loss of motivation, and cognitive fatigue (Ceban et al., 2022; Nakagawa et al., 2016).

Furthermore, altered FC between the DMN and the insular cortex reflects disruptions in the ventral attention and salience networks in the CRPs, providing an anatomical substrate for complaints regarding “brain fog”. Atrophy in the subcortical (thalamus, amygdala, hippocampus) and cortical (prefrontal cortex, anterior cingulate cortex) limbic nodes, coupled with microstructural damage to the UF and cingulum bundle, may underlie the high prevalence of memory impairment in CRPs. We also observed structural and functional disruptions in the caudate nucleus, which is known to facilitate slow-wave rapid eye movement (REM) sleep (Jones & Ikuta, 2022). These aberrations could provide a possible explanation for reports of unrefreshing sleep (27/59) in CRPs. Overall, this concordance between neuroanatomical findings and behavioral reports reinforces a unified model whereby SARS-CoV-2 preferentially injures limbic–basal ganglia circuits, yielding the multidomain symptomatology of post-COVID syndrome.

## 5 Limitations

Certain limitations of this study need to be acknowledged to contextualize the findings and inform future research directions. One key limitation is the absence of comprehensive pre-infection medical histories and detailed comorbidity data for both CRPs and HCs. Pre-existing conditions such as hypertension, diabetes, or respiratory illnesses, known to influence neurological health, could have contributed to the observed differences in brain structure and connectivity. While individuals with a history of neurological disorders or brain trauma were excluded to minimize confounding factors, subtle baseline health differences may still have influenced the results. In addition, the data were acquired in 2021, when vaccination in India had only recently begun and coverage was limited. While it was reasonable to assume that the recruited patients in this study were likely unvaccinated at the time of infection, it is worth noting that vaccination status may influence both disease severity and long-term neurological outcomes. Future studies incorporating pre-infection medical records, vaccination details, and additional control groups with other viral illnesses will be essential to disentangle the effects of COVID-19 from underlying health conditions.

The statistical power of our findings is also limited by the relatively small cohort as compared with many large-scale studies (e.g. Douaud et al., 2022). In addition, the cross-sectional nature of our study limits our understanding of the long-term evolution of post-COVID-19 effects on the brain (e.g., whether the changes are persistent or whether they reverse over time). Future studies with longitudinal data could help monitor these changes in CRPs over time.

Another potential limitation is the risk of undetected asymptomatic subjects with COVID-19 infections within the HC group. Given the high prevalence of asymptomatic cases during the pandemic and the limitations of testing at the time, it is possible that some control subjects had prior, unconfirmed SARS-CoV-2 infections. Although rigorous screening criteria were applied, including negative RT-PCR confirmation for symptomatic individuals, asymptomatic infections could have reduced the contrast between groups. Future studies employing serological testing or antibody assays may help better classify subjects and refine group comparisons.

While our HC and CRP cohorts are matched in terms of age and sex, stratification of CRPs based on infection severity resulted in an age imbalance between NHPs and HPs. There is a difference of over a decade between the ages of hospitalized patients (39.48 ± 11.95 years) and non-hospitalized patients (27.30 ± 7.72 years). While we tried to reduce the effect of age-related changes in FC by adding age as a covariate of no interest, some residual effect of age-related FC decline may have influenced our results. In addition, the HC, NHP, and HP groups differed in size, with the HP group being the smallest. To mitigate potential bias from unequal group sizes, we used non-parametric permutation-based testing (FSL’s randomize) with 5000 permutations and TFCE correction, which is robust to unequal group sizes and variance heterogeneity. While these steps help reduce the risk of spurious findings, the smaller HP sample would limit statistical power and may lead to underestimation of the true extent of connectivity alterations. A similar imbalance in sizes of the HC and CRP groups also appears in the analysis of CBF using ASL images (67 CRPs and 47 HCs) and would limit statistical power. Another imbalance in our cohort is that of sex within the HC and CRP cohorts. While the groups were sex matched, there was an over-representation of males in the studied sample. Despite our efforts at covariate adjustment, the sex imbalance could still have influenced findings, particularly in subcortical structures. Future studies with comparable groups and well-matched severity stratification could help distill the impact of COVID-19 on FC and blood perfusion from spurious effects.

We were able to collect self-reported data regarding the post-COVID symptoms from 59 CRPs. However, it is important to note that these are not an objective assessment of cognitive or behavioral deficits in CRPs. Therefore, while our neuroimaging findings in limbic–basal ganglia circuits plausibly relate to symptoms such as fatigue, attentional deficits, and insomnia, we could not quantitatively test the correlations of these markers with post-COVID symptoms. Future studies combining imaging with standardized behavioral and neuropsychological assessments could address this limitation.

## 6 Conclusion

This multimodal MRI study of 76 COVID-19 survivors and 51 matched controls reveals that long-term neurological sequelae concentrate in interconnected limbic–basal ganglia circuits rather than manifesting as diffuse brain injury. We observed focal subcortical atrophy (thalamus, hippocampus, amygdala, putamen, caudate, pallidum), paralimbic cortical thinning, and microstructural disruption of the uncinate fasciculus and cingulum bundle. Resting-state analyses showed severity-dependent decoupling between default mode and salience networks, while perfusion deficits normalized by 3–6 months post-infection. Overall, our findings help identify neuroimaging biomarkers for the post-COVID syndrome and underscore the need for targeted investigations. These circuit-specific alterations could be related to patients’ persistent fatigue, memory impairments, attentional lapses, and sleep disturbance. Future investigations could utilize the neuroimaging markers identified here and study their correlation with scores in cognitive, neuropsychological, or sleep tests in CRPs, helping build a unified model of post-COVID neuropathophysiology. Additionally, longitudinal work integrating high-field MRI, PET microglial markers, and fluid biomarkers will be essential to chart recovery trajectories and optimize personalized rehabilitation strategies.

## Data Availability

The source data are available upon reasonable request from the corresponding author. All computations were conducted with pre-existing FSL, SPM, FreeSurfer, and MATLAB toolboxes along with Python packages as described in Section 2. The processed data for each modality and statistical analysis scripts are available at https://osf.io/ua6r3/?view_only=6ce66b5eff484a7fa282b6c23f6f9234.
